# Supplementary material for: In-plane reorientation induced single laser pulse magnetization reversal
Source: Nat Commun. 2023 Aug 17;14:5000. doi: 10.1038/s41467-023-40721-z (PMC10435580; doi:10.1038/s41467-023-40721-z)
Supplement: Supplementary file 1 — Supplementary Information [file 41467_2023_40721_MOESM1_ESM.pdf]

In-plane reorientation induced single laser pulse magnetization reversal

Supplementary information

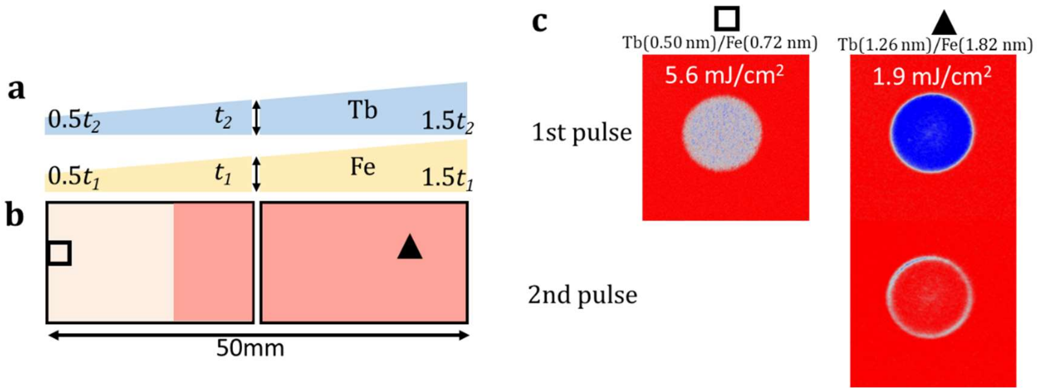

Supplementary figure 1: Single pulse switching in [Tb(wedge)/Fe(wedge)]$_4$ multilayer. a Sketch of the multilayer and wedge of Tb and Fe thicknesses, the Fe thickness in the middle is t_1 = 1.3 nm, while the Tb thickness in the middle t_2 =0.9 nm. The thickness ratio of Fe and Tb is constant and equals to 1.44 everywhere. b Light red color shows the region of Tb and Fe thicknesses (1.12 nm < t_{Fe} < 1.96 nm, 0.77nm < t_{Tb} < 1.36 nm) where single shot switching occurs. c The response to single laser pulse at different positions (pulse duration: 50 fs).

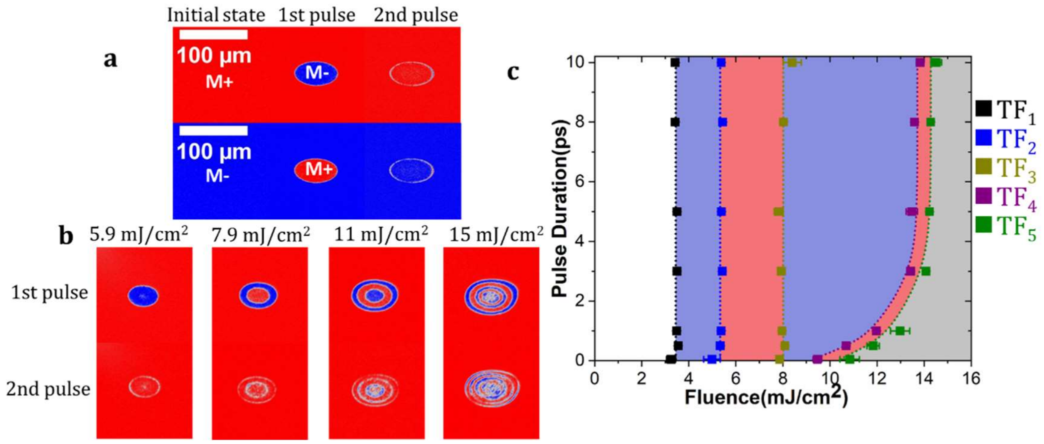

Supplementary figure 2-a. Single switching and state diagram in [Tb(1.06 nm)/Co(1.78 nm)]$_5$ multilayer. a Background subtracted images after each single pulse with 5.1 mJ/cm^2 laser pulse at 50fs. b Background subtracted images after first single with 50 fs laser pulse of different fluence. c State diagram pulse duration versus laser fluence. TF1 is the border for switching; TF2 is the border to two domains state, one ring; TF3 is the border to three domains state, two rings; TF4 is the border to four domains state, three rings; TF5 is the border to multidomain state at the center and four rings. The dotted lines are guide for the eyes.

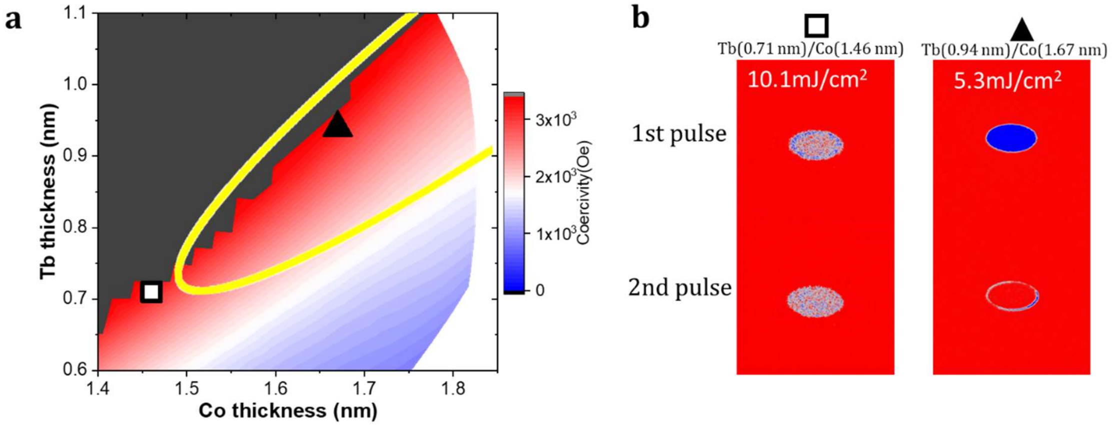

Supplementary figure 2-b. Single switching in [Tb(wedge)/Co(wedge)]$_5$ cross-wedge multilayer. a Coercivity map of [Tb/Co]$_5$ along the Tb and Co thickness wedges. The grey color indicates the region in which the applied field was not sufficient to saturate the stack. The region shown by the solid yellow line indicates the Tb and Co thicknesses region where single switching occurs. b Background subtracted images achieved using 50 fs laser pulse at two positions enclosed by the symbols in a.

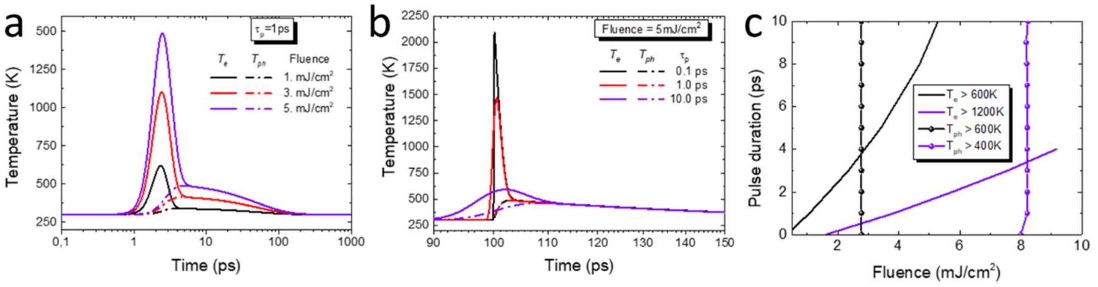

Supplementary figure 3. Time evolution of the T_e and T_{ph}, according with 2T mode. a for constant pulse duration of 1ps and variable fluence. b for constant fluence of 5.0 mJ/cm^2 and variable pulse duration respectively. Simulation parameters: $t_{FM} = 100\ nm$, $T_0 = 300K$, $\mathrm{G} = 2.5 \cdot\ 10^{17}\ \mathrm{W} \cdot m^{-3}K^{-1}$, $C_e = \gamma T_e = 225\ Jm^{-3}K^{-2}Te$ and $C_{ph} = 2.6 \cdot 10\ Jm^{-3}K^{-1}$ [25,29]. c The fluence threshold F_{th} per pulse duration in order to reach $T_e > 1200K, 600K$ (black and violet lines respectively) and $T_{ph} > 600K, 400K$ (black and violet dotted lines respectively).

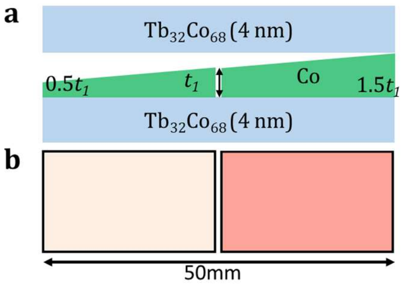

Supplementary figure 4-a. Single switching in $Tb_{32}Co_{68}$/Co(wedge)/$Tb_{32}Co_{68}$ trilayer. a Simple stack and wedge of Co thickness description, where the Co thickness in the middle t_1 = 1.5 nm. The thickness of Co varies from 0.83 nm (left) to 2.27 nm (right). b Light red color shows the region of Co thickness (1.52 nm < t_{Co} < 2.27 nm) where single shot switching occurs in $Tb_{32}Co_{68}$/Co/ $Tb_{32}Co_{68}$ trilayer.

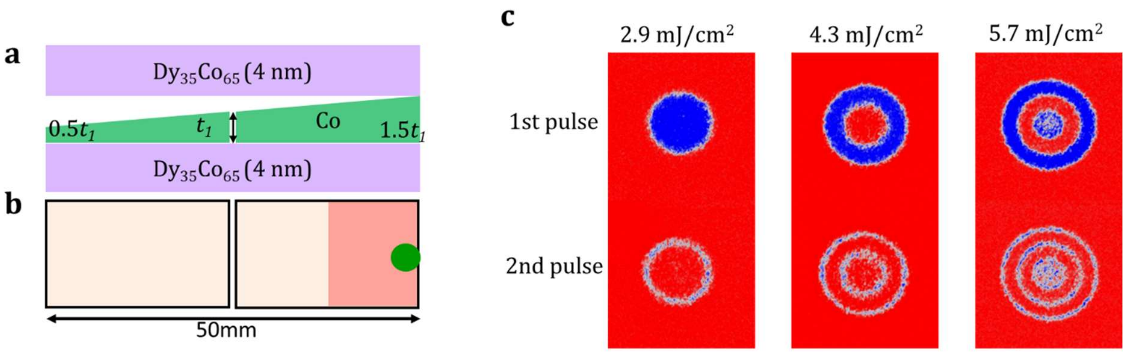

Supplementary figure 4-b. Single switching in $Dy_{35}Co_{65}$/Co(wedge)/$Dy_{35}Co_{65}$ trilayer. a Simple stack and wedge of Co thickness description, where the Co thickness in the middle t_1 = 2 nm. The thickness of Co varies from 1.02 nm (left) to 3.02 nm (right). b Light red color shows the region of Co thickness (2.41 nm < t_{Co} < 3.02 nm) where single shot switching occurs in $Dy_{35}Co_{65}$/Co/$Dy_{35}Co_{65}$trilayer; c Background subtracted images after each 50fs laser pulse with different fluences, where t_{Co} = 2.98 nm.

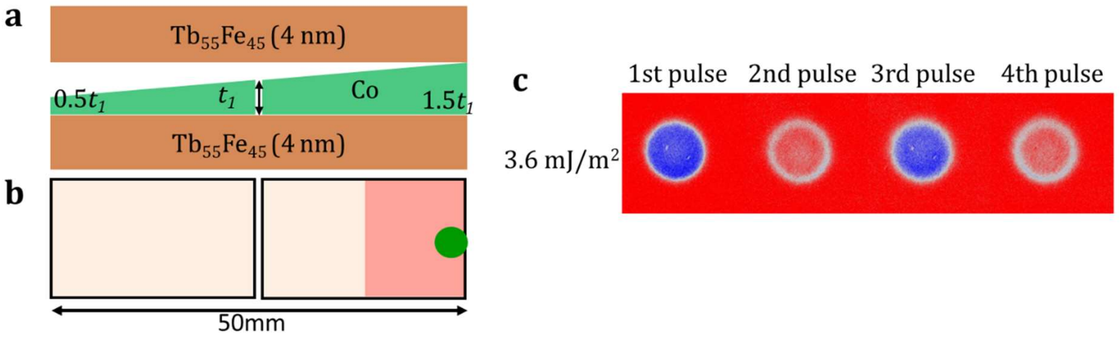

Supplementary figure 4-c. Single switching in $Tb_{55}Fe_{45}$(4 nm)/Co(wedge)/$Tb_{55}Fe_{45}$ (4 nm) trilayer by 50 fs single pulse. a Simple stack and wedge of Co thickness description, where the Co thickness in the middle t_1 = 1.5 nm. The thickness of Co varies from 0.83 nm (left) to 2.27 nm (right). b Light red color shows the region of Co thickness (3.89 nm < t_{Co} < 5.60 nm) where single pulse switching occurs. c Background subtracted images after each 50fs laser pulse and fluence of 3.6 mJ/cm^2 measured at the green point position with t_{Co} = 2.27 nm

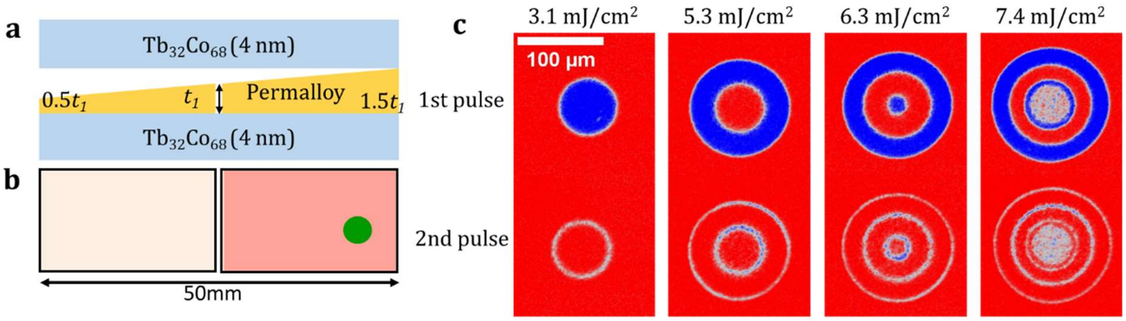

Supplementary figure 4-d. Single switching in $Tb_{32}Co_{68}$/Py(wedge)/$Tb_{32}Co_{68}$ trilayer. a Simple stack and wedge of Py thickness description, where the Py thickness in the middle t_1 = 3.7 nm. The thickness of Py varies from 2.05 nm (left) to 5.60 nm (right). b Light red color shows the region of Py thickness (3.89 nm < t_{Py} < 5.60 nm) where single shot switching occurs. c Background subtracted images after each 50fs laser pulse with different fluences measured at the green point position with t_{Py} = 5.10 nm.

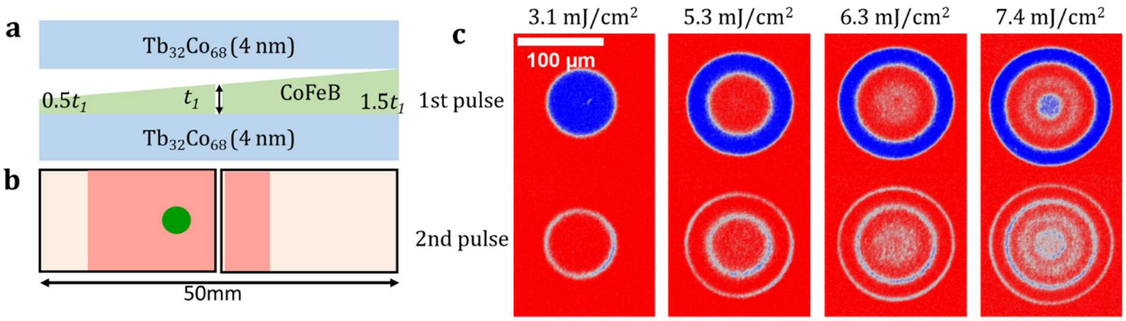

Supplementary figure 4-e. Single switching in $Tb_{32}Co_{68}$/CoFeB(wedge)/$Tb_{32}Co_{68}$ trilayer. a Simple stack and wedge of CoFeB thickness description, where the CoFeB thickness in the middle t_1 = 2.5 nm. The thickness of CoFeB varies from 1.39 nm (left) to 3.78 nm (right). b Light red color shows the region of CoFeB thickness (1.67 nm < t_{CoFeB} < 2.87 nm) where single shot switching occurs. c Background subtracted images after each 50fs laser pulse with different fluences measured at the green point position with t_{CoFeB} = 3.33 nm.

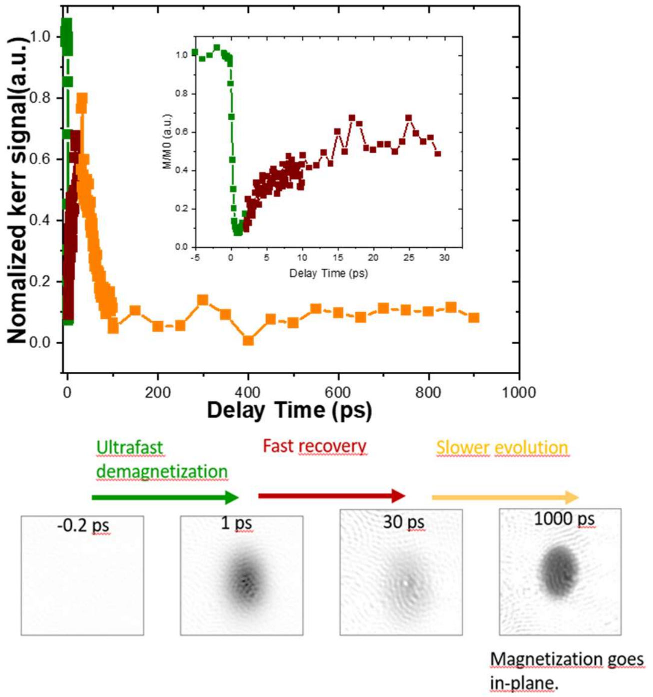

Supplementary figure 5: Demagnetization dynamics in [Tb(1.06 nm)/Co(1.78 nm)]$_5$ multilayer. The Kerr images show that magnetization decrease is not due to the appearance of a domain pattern. The region of interest to plot the curve is 2 μm in diameter.

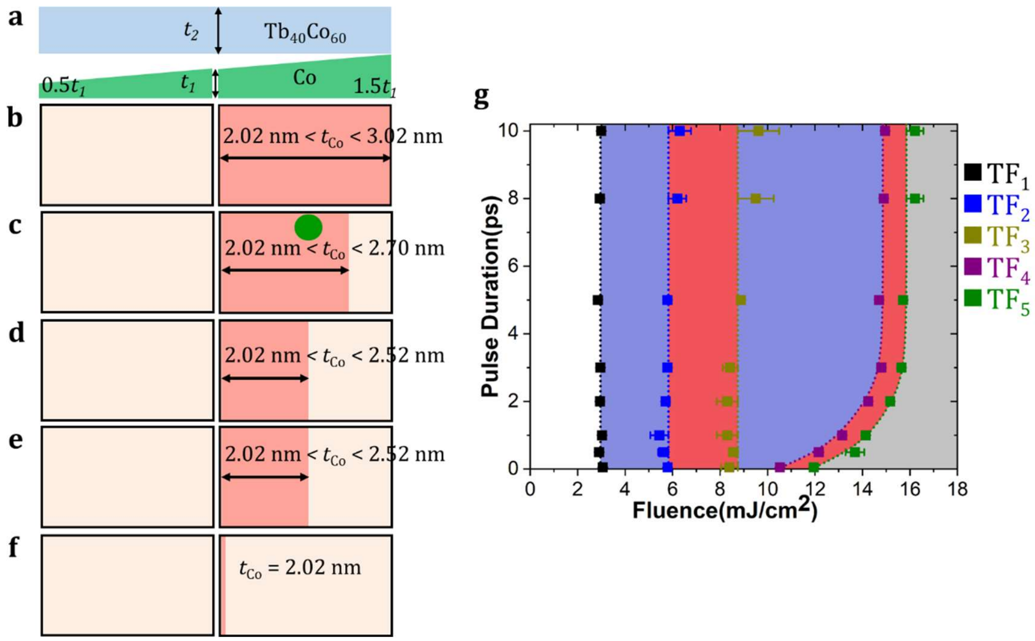

Supplementary figure 6. Single pulse switching in [$Co_{60}Tb_{40}$)(x)/Co(wedge)]$_3$, x varying from 1 to 5nm. Thickness mapping of Co layer that show single switching in [$Tb_{40}Co_{60}$/Co]$_3$ multilayer. a Description of Co wedge. The thickness of Co layer is a wedge where the thickness in the middle t_1 = 2 nm. b-f Light red color shows the region where single switching occurs when the thickness of TbCo layer is b t_2 = 5 nm; c t_2 = 4 nm; d t_2 = 3 nm; e t_2 = 2 nm; f t_2 = 1 nm. g State diagram obtained at green round position in c), where t_{Co} = 2.52 nm.

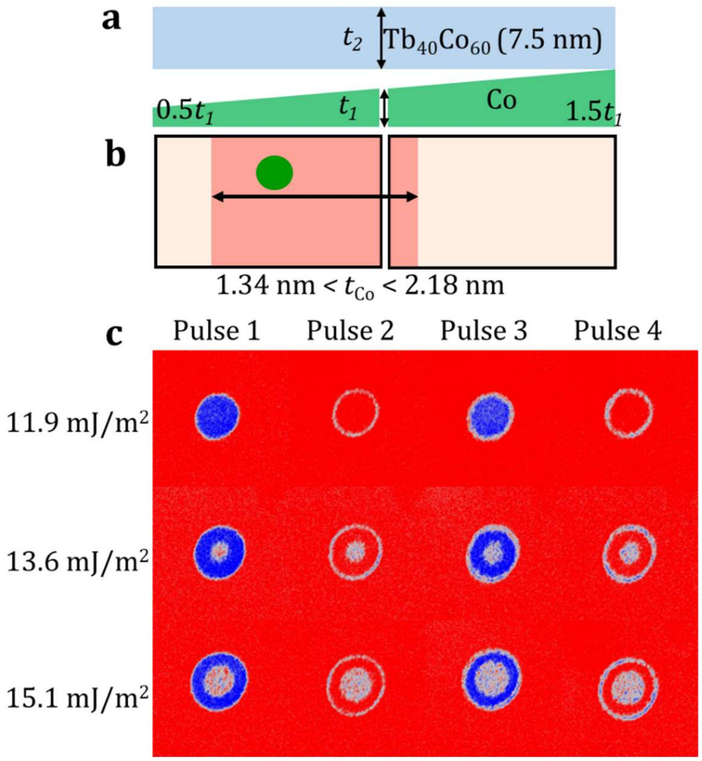

Supplementary figure 7. Single pulse switching in [$Tb_{40}Co_{60}$)(7.5 nm)/Co(wedge)$]_3$ multilayer. a Simple stack and wedge of Co thickness description, where the Co thickness in the middle t_1 = 2 nm. The thickness of Co varies from 1.11 nm (left) to 3.02 nm (right). b Light red color shows the region of Co thickness (1.34 nm < t_{Co} < 2.18 nm) where single shot switching occurs. c Background subtracted images after each 50fs laser pulse with different fluences obtained at green round position in b, where t_{Co} = 1.57 nm.

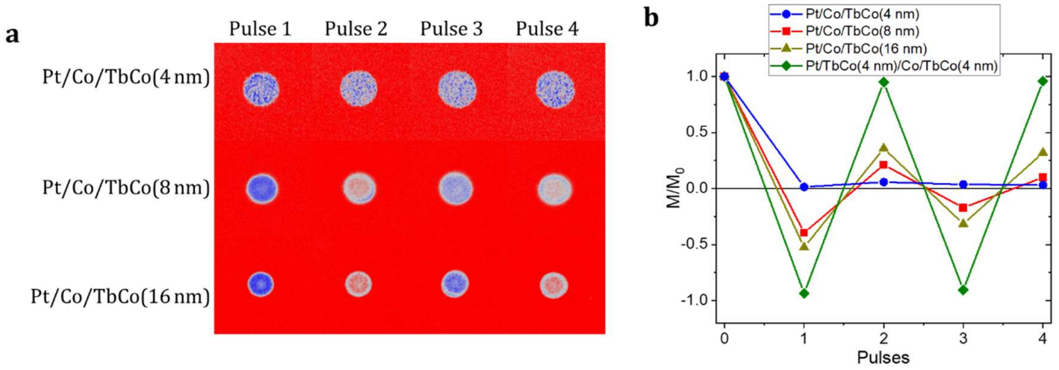

Supplementary figure 8. a Single pulse switching in Co(2.27 nm)/$Tb_{32}Co_{68}$ (x) bilayer where x is 4 nm (top), 8 nm (middle) and 16 nm (bottom). b The changes of magnetization around the center of the spot, estimated from magnetic contrast, are plotted as a function of number of pulses. The value of $Tb_{32}Co_{68}$ (4 nm) /Co(2.27 nm)/$Tb_{32}Co_{68}$ (4 nm) is given for comparison.

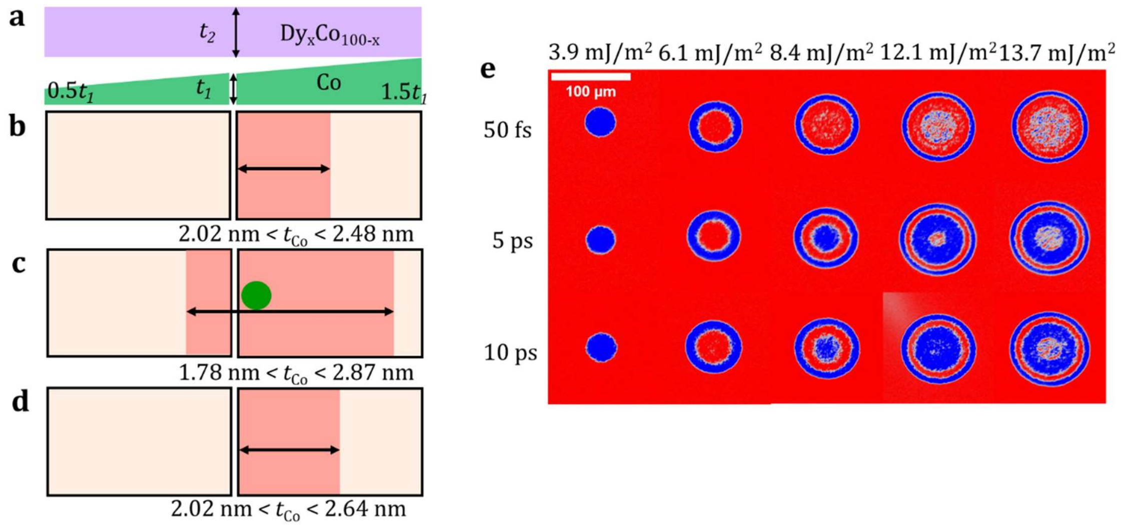

Supplementary figure 9: Single pulse reversal in $[Dy_{1-y}Co_y(4\ nm)/Co(wedge)]_3$ for y = 65, 70 and 75%. Thickness mapping of Co layer that show single switching. a Description of Co wedge. The thickness of Co layer is a wedge where the thickness in the middle t_1 = 2 nm. The thickness of DyCo alloy layer is kept constant at t_2 = 4 nm. Light red color shows the region of Co thickness where single shot switching occurs in b $Dy_{35}Co_{65}$; c $Dy_{30}Co_{70}$; d $Dy_{25}Co_{75}$. e State diagram obtained at green round position in **c**, where t_{Co} = 2.10 nm.

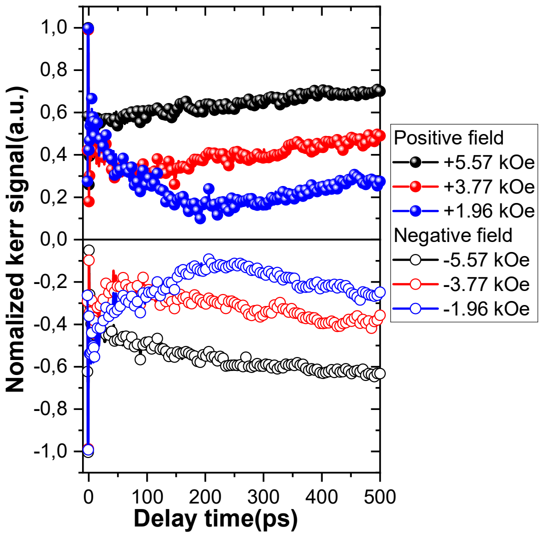

Supplementary figure 10. Dynamics of a $[Co(2.1\ nm)/(Co_{60}Tb_{40})(4)]_3$ multilayer. The dynamics are recorded using different applied fields in both opposite directions. In all those measurements, the fluence was kept constant at 5.0 mJ/cm^2.

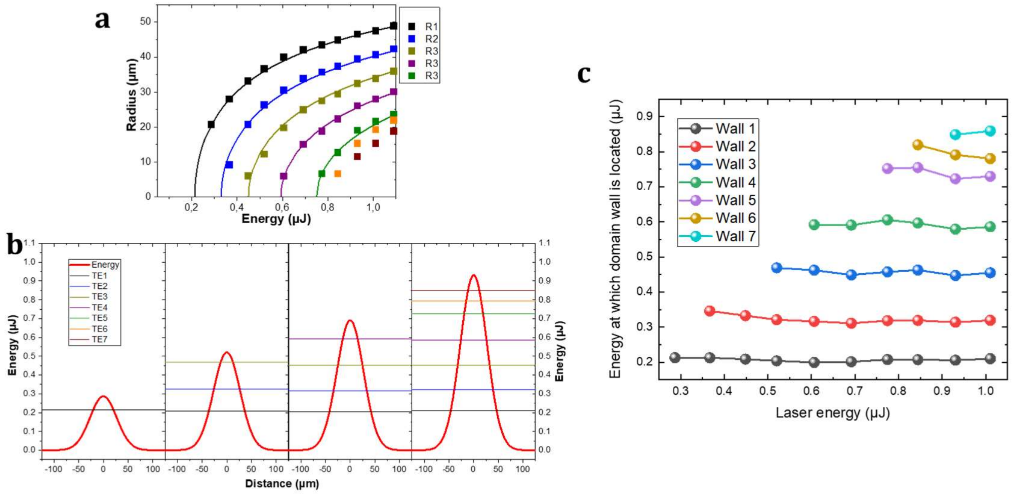

Supplementary figure 11. Analysis of the domain wall position of the ring structure, in space and energy, versus laser pulse energy in $Tb_{32}Co_{67}$(4 nm)/Co(1.52 nm)/$Tb_{32}Co_{67}$(4 nm) trilayer. a Ring radius versus energy. b Plot of the gaussian laser profile for different laser energies and report of the ring radius. c Local energy of the domain walls versus total laser energy.

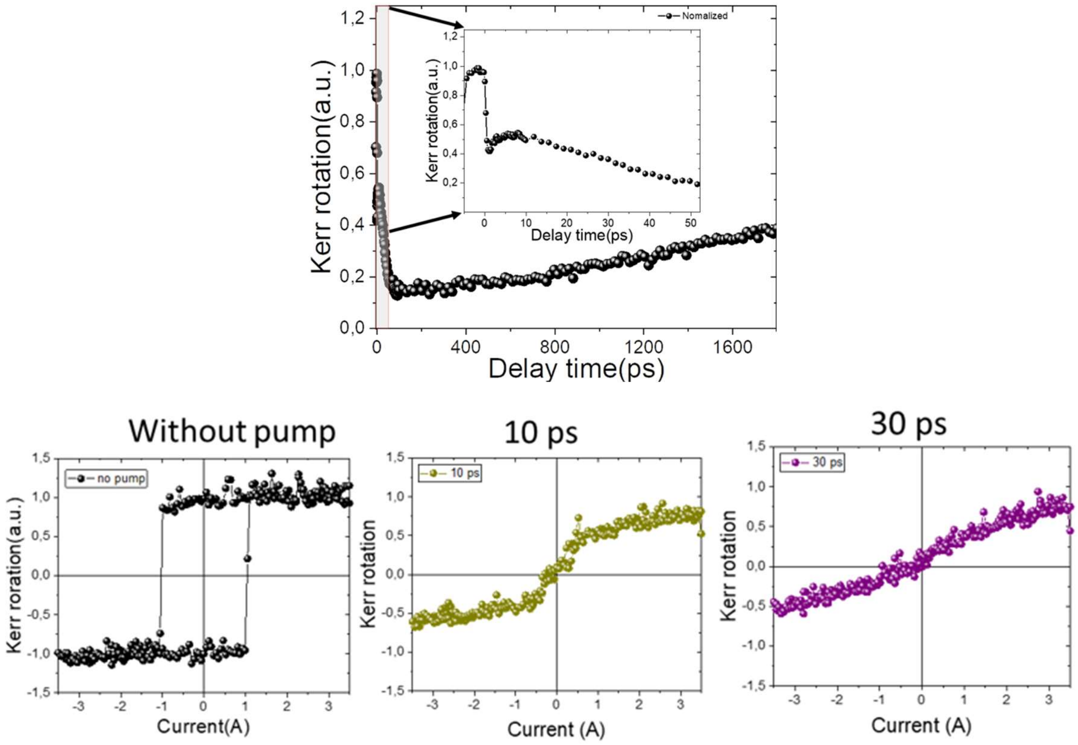

Supplementary figure 12. Hysteresis loops measured by pump-probe method at different delay time with pump fluence of 3.3 mJ/cm^2 for a sample with the composition TbCo(4)/Co(2)/TbCo(4)

Stacks	Composition	Field (Oe)	Fluence(mJ/cm^2)	Repetition rate(kHz)
$[Tb/Co]_5$ multilayer	t(Tb) = 1.06 nm<br>t(Co) = 1.78 nm	550	6.32	0.2
$[TbCo/Co]_3$ multilayer	$t(Tb_{40}Co_{60})$ = 4 nm<br>t(Co) = 2.10 nm	610	6.52	10
$[DyCo/Co]_3$ multilayer	$t(Dy_{30}Co_{70})$ = 4 nm<br>t(Co) = 2.18 nm	570	3.83	1
TbCo/Co/TbCo trilayer	$t(Tb_{32}Co_{68})$ = 4 nm<br>t(Co) = 2.24 nm	690	3.26	1

Supplementary table 1. Stacks and TR-MOKE conditions corresponding to Figure 4. The repetition rate has been optimized to get the best contrast without changing the properties of the layers (annealing or burning).

References

1. Katine, J. A., Albert, F. J., Buhrman, R. A., Myers, E. B. & Ralph, D. C. Current-Driven Magnetization Reversal and Spin-Wave Excitations in Co/Cu/Co Pillars. Phys. Rev. Lett. 84, 3149 (2000).
2. Miron, I. M., Garello, K., Gaudin, G., Zermatten, P.-J., Costache, M. V., Auffret, S. Bandiera, S., Rodmacq, B., Schuhl, A. & Gambardella, P. Perpendicular switching of a single ferromagnetic layer induced by in-plane current injection. Nature 476, 189–193 (2011).
3. Liu, H., Bedau, D., Backes, D., Katine, J., Langer, J. & Kent, A. Ultrafast switching in magnetic tunnel junction based orthogonal spin transfer devices. Appl. Phys. Lett. 97, 242510 (2010).
4. Garello, K., Avci, C. O., Miron, I. M., Baumgartner, M., Ghosh, A., Auffret, S., Boulle, O., Gaudin, G. & Gambardella, P. Ultrafast magnetization switching by spin-orbit torques. Appl. Phys. Let. 105, 212402 (2014).
5. Beaurepaire, E., Merle, J.-C., Daunois, A. & Bigot, J.-Y. Ultrafast spin dynamics in ferromagnetic nickel. Phys. Rev. Lett. 76, 4250 (1996).
6. Stanciu, C. D., Hansteen, F., Kimel, A. V., Kirilyuk, A., Tsukamoto, A., Itoh, A. & Rasing, Th. All-Optical Magnetic Recording with Circularly Polarized Light. Phys. Rev. Lett. 99, 047601 (2007).
7. Radu, I., Vahaplar, K., Stamm, C., Kachel, T., Pontius, N., Durr, H. A., Ostler, T. A., Barker, J., Evans, R. F. L., Chantrell, R. W., Tsukamoto, A., Itoh, A., Kirilyuk, A., Rasing, T. & Kimel, A. V. Transient ferromagnetic-like state mediating ultrafast reversal of antiferromagnetically coupled spins. Nature 472, 205-209 (2011).
8. Ostler, T. A., Barker, J., Evans, R. F. L., Chantrell, R. W., Atxitia, U., Chubykalo-Fesenko, O., El Moussaoui, S., Le Guyader, L., Mengotti, E., Heyderman, L. J., Nolting, F., Tsukamoto, A., Itoh, A., Afanasiev, D., Ivanov, B. A., Kalashnikova, A. M., Vahaplar, K., Mentink, J., Kirilyuk, A. & Kimel, A. V. Ultrafast heating as a sufficient stimulus for magnetization reversal in a ferrimagnet. Nature Communications 3, 666 (2012).
9. Steil, D., Alebrand, S., Hassdenteufel, A., Cinchetti, M. & Aeschlimann, M. All-optical magnetization recording by tailoring optical excitation parameters. Phys. Rev. B 84, 224408 (2011).
10. Gorchon, J., Wilson, R. B., Yang, Y., Pattabi, A., Chen, J. Y., He, L., Wang, J. P., Li, M. & Bokor, J. Role of electron and phonon temperatures in the helicity-independent all-optical switching of GdFeCo. Phys. Rev. B 94, 184406 (2016).
11. Yang, Y., Wilson, R. B., Gorchon, J., Lambert, C.-H., Salahuddin, S. & Bokor, J. Ultrafast magnetization reversal by picosecond electrical pulses. Science Advances 3, e1603117 (2017).
12. Davies, C. S., Janssen, T., Mentink, J. H., Tsukamoto, A., Kimel, A. V., Van Der Meer, A. F. G., Stupakiewicz, A. & Kirilyuk A. Pathways for Single-Shot All-Optical Switching of Magnetization in Ferrimagnets. Phys. Rev. Appl. 13, 024064 (2020).
13. Wei, J., Zhang, B., Hehn, M., Zhang, W., Malinowski, G., Xu, Y., Zhao, W. & Mangin, S. All-Optical Helicity-Independent Switching state diagram in GdFeCo alloys. Phys. Rev. Appl. 15, 054065 (2021).
14. Lalieu, M. L., Peeters, M. J. G., Haenen, S. R. R., Lavrijsen, R. & Koopmans, B. Deterministic all-optical switching of synthetic ferrimagnets using single femtosecond laser pulses. Phys. Rev. B 96, 220411 (2017).
15. Zhang, W., Lin, J. X., Huang, T. X., Malinowski, G., Hehn, M., Xu, Y., Mangin, S. & Zhao, W. Role of spin-lattice coupling in ultrafast demagnetization and all optical helicity-independent single-shot switching in $Gd_{1-x-y}Tb_yCo_x$ alloys. Phys. Rev. B 105, 054410 (2022).
16. Banerjee, C., Teichert, N., Siewierska, K. E., Gercsi, Z., P. Atcheson, G. Y., Stamenov, P., Rode, K., Coey, J. M. D. & Besbas, J. Single pulse all-optical toggle switching of

magnetization without gadolinium in the ferrimagnet Mn2RuxGa. Nature Communications 11, 4444 (2020).
17. Atxitia, U., Barker, J. & Chantrell, R. W. Controlling the polarity of the transient ferromagneticlike state in ferrimagnets. Phys. Rev. B 89, 224421 (2014).
18. Davies, C. S., Bonfiglio, G., Rode, K., Besbas, J., Banerjee, C., Stamenov, P., Coey, J. M. D., Kimel, A. V. & Kirilyuk, A. Exchange-driven all-optical magnetic switching in compensated 3d ferrimagnets. Phys. Rev. Research 2, 032044 (2020).
19. Avilés-Félix, L., Álvaro-Gómez, L., Li, G., Davies, C. S., Olivier, A., Rubio-Roy, M., Auffret, S., Kirilyuk, A., Kimel, A. V., Rasing, T., Buda-Prejbeanu, L. D., Sousa, R. C., Dieny, B. & Prejbeanu, I. L. Integration of Tb/Co multilayers within optically switchable perpendicular magnetic tunnel junctions. AIP Advances 9, 125328 (2019).
20. Avilés-Félix, L., Olivier, A., Li, G., Davies, C. S., Álvaro-Gómez, L., Rubio-Roy, M., Auffret, S., Kirilyuk, A., Kimel, A. V., Rasing, T., Buda-Prejbeanu, L. D., Sousa, R. C., Dieny, B. & Prejbeanu, I. L. Single-shot all-optical switching of magnetization in Tb/Co multilayer-based electrodes. Scientific Reports 10, 5211 (2020).
21. Kichin, G., Hehn, M., Gorchon, J., Malinowski, G., Hohlfeld, J. & Mangin, S. From Multiple- to Single-Pulse All-Optical Helicity-Dependent Switching in Ferromagnetic Co/Pt Multilayers. Phys. Rev. Appl. 12, 024019 (2019).
22. Davies, C. S., Prabhakara, K. H., Davydova, M. D., Zvezdin, K. A., Shapaeva, T. B., Wang, S., Zvezdin, A. K., Kirilyuk, A., Rasing, Th. & Kimel, A. V. Anomalously Damped Heat-Assisted Route for Precessional Magnetization Reversal in an Iron Garnet. Phys. Rev. Lett. 122, 027202 (2019).
23. Shelukhin, L. A., Pavlov, V. V., Usachev, P. A., Shamray, P. Yu., Pisarev, R. V. & Kalashnikova, A. M. Ultrafast laser-induced changes of the magnetic anisotropy in a low-symmetry iron garnet film. Phys. Rev. B 97, 014422 (2018).
24. Kats, V. N., Linnik, T. L., Salasyuk, A. S., Rushforth, A. W., Wang, M., Wadley, P., Akimov, A. V., Cavill, S. A., Holy, V., Kalashnikova, A. M. & Scherbakov, A. V. Ultrafast changes of magnetic anisotropy driven by laser-generated coherent and noncoherent phonons in metallic films. Phys. Rev. B 93,214422 (2016).
25. Moreno, R., Ostler, T. A., Chantrell, R. W. & Chubykalo-Fesenko, O. Conditions for thermally induced all-optical switching in ferrimagnetic alloys: Modeling of TbCo. Phys. Rev. B 96, 014409 (2017).
26. Yamauchi, K., Habu, K. & Sato, N. Magnetic structure of Tb‐Fe films with an artificially layered structure. Journal of Applied Physics 64, 5748 (1988).
27. Hansen, P., Clausen, C., Much, G., Rosenkranz, M. & Witter, K. Magnetic and magneto‐optical properties of rare‐earth transition‐metal alloys containing Gd, Tb, Fe, Co. J. Appl. Phys. 66, 756 (1989).
28. Hu, Z., Besbas, J., Smith, R., Teichert, N., Atcheson, G., Rode, K., Stamenov, P. & Coey, J. M. D. Single-pulse all-optical partial switching in amorphous Dy_xCo_{1-x} and Tb_xCo_{1-x} with random anisotropy. Appl. Phys. Lett. 120, 112401 (2022).
29. Frietsch, B., Donges, A., Carley, R., Teichmann, M., Bowlan, J., Döbrich, K., Carva, K., Legut, D., Oppeneer, P. M., Nowak, U. & Weinelt, M. The role of ultrafast magnon generation in the magnetization dynamics of rare-earth metals. Sci. Adv. 6, eabb1601 (2020).
30. Mishra, K., Blank, T. G. H., Davies, C. S., Avilés-Félix, L., Salomoni, D., Buda-Prejbeanu, L. D., Sousa, R. C., Prejbeanu, I. L., Koopmans, B., Rasing, Th., Kimel, A. V. & Kirilyuk, A. Dynamics of all-optical single-shot switching of magnetization in Tb/Co multilayers, Phys. Rev. Research 5, 023163 (2023).
